# Regulation of the Ras-MAPK and PI3K-mTOR Signalling Pathways by Alternative Splicing in Cancer

**DOI:** 10.1155/2013/568931

**Published:** 2013-09-03

**Authors:** Zahava Siegfried, Serena Bonomi, Claudia Ghigna, Rotem Karni

**Affiliations:** ^1^Department of Biochemistry and Molecular Biology, The Institute for Medical Research Israel-Canada, Hebrew University-Hadassah Medical School Ein Kerem, 91120 Jerusalem, Israel; ^2^Institute of Molecular Genetics, National Research Council (IGM-CNR), Via Abbiategrasso 207, 27100 Pavia, Italy

## Abstract

Alternative splicing is a fundamental step in regulation of gene expression of many tumor suppressors and oncogenes in cancer. Signalling through the Ras-MAPK and PI3K-mTOR pathways is misregulated and hyperactivated in most types of cancer. However, the regulation of the Ras-MAPK and PI3K-mTOR signalling pathways by alternative splicing is less well established. Recent studies have shown the contribution of alternative splicing regulation of these signalling pathways which can lead to cellular transformation, cancer development, and tumor maintenance. This review will discuss findings in the literature which describe new modes of regulation of components of the Ras-MAPK and PI3K-mTOR signalling pathways by alternative splicing. We will also describe the mechanisms by which signals from extracellular stimuli can be communicated to the splicing machinery and to specific RNA-binding proteins that ultimately control exon definition events.

## 1. Introduction

In the past several decades cancer research has focused on genetic alterations such as mutations, copy number variations, and translocations that are detected in malignant tissues and contribute to the initiation and progression of cancer. In recent years it is becoming clear that epigenetic changes, including transcriptional and posttranscriptional alterations, also play a major role in cancer development and thus should be the direction of future research [1–4]. Mutations and copy number variations in splicing regulators have been identified in several types of cancer, supporting the notion that changes in splicing fidelity contribute to cancer development [5–9].

Alternative splicing plays a major role in cancer development and progression as many tumor suppressors and oncogenes are modulated by alternative splicing [10, 11]. However, the role of alternative splicing regulators in cancer development is mostly unknown, and only recently the first direct evidence for an oncogenic role of a splicing factor has been shown [9, 12–15]. 

 The Ras-MAPK and PI3K-mTOR signalling pathways are deregulated in many cancers by genetic and epigenetic aberrations [16–18]. Several key components in these pathways, such as Ras, B-RAF, C-RAF, MEK1, PI3K, and Akt, are activated by mutations or gene amplifications, while other components that inhibit these pathways, such as PTEN, LKB1, and TSC1/2, are inactivated by genomic deletions and mutations [16–20]. Pharmacological inhibitors of enzymes in these pathways, such as BRAF inhibitors and mTOR inhibitors, are already being used in clinical settings to treat cancer, while others (PI3K and MEK1 inhibitors) are in advanced stages of clinical trials [21–26]. Although the Ras-MAPK and PI3K-mTOR pathways are at the center of intensive research, and many genetic alterations that activate or inactivate these pathways have been discovered, much less is known about the epigenetic and posttranscriptional regulation of these signalling pathways. Recent studies have revealed how these pathways can be regulated by alternative splicing and by splicing regulators and are the focus of this review.

 Here, we discuss the intricate relationship between alternative splicing and signalling at different levels: (i) how the activity of components in the Ras-MAPK signalling pathway is regulated by alternative splicing in cancer cells; (ii) how the activity of components in the PI3K-mTOR pathway is regulated by alternative splicing in cancer cells; (iii) mechanisms by which extracellular stimuli can be communicated to the splicing machinery and to specific RNA-binding proteins that ultimately control exon definition events.

 Alternative splicing can affect the activity of signalling effectors contributing to their constitutive (or improper) function. The most well-characterized examples are represented by members of the receptor tyrosine kinase (RTK) family; EGFR, FGFR, INSR, VEGFR, MET, and Ron [2, 19, 27–39]. In addition, recent studies have also focused on members of non-receptor cytosolic protein kinases, such as Src, Ras, and Raf and on non-kinase cytosolic receptors, including androgen and estrogen receptors [20, 40–43] (Table 1).

## 2. Regulation of the Ras-MAPK Pathway by Alternative Splicing

**Figure 1:**
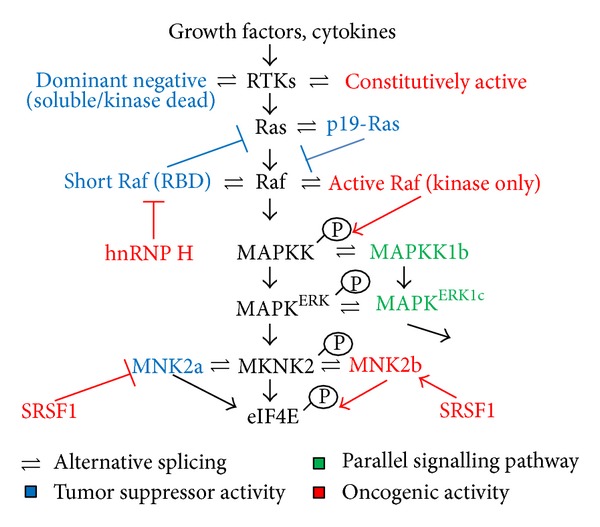
Alternative splicing regulation of the Ras-MAPK pathway. Growth factors and cytokines activate receptor tyrosine kinases (RTKs) which in turn lead to activation of Ras. RTKs can be alternatively spliced to generate soluble truncated isoforms which act in a dominant-negative manner or constitutively active isoforms which are active regardless of ligand binding. Ras can be alternatively spliced to generate p19-Ras which cannot activate its downstream effector Raf. GTP-bound p21-Ras activates A-, B-, and C-Raf which can be alternatively spliced to generate inactive dominant-negative isoforms containing only the Ras binding domain (RBD) or constitutively active isoforms containing only the kinase domain. Oncogenic splicing factor hnRNP H inhibits the production of dominant-negative A-Raf isoforms. Raf phosphorylates MAPKK (MEK) which in turn phosphorylates MAPK-ERK. MAPKK1 and ERK1 can generate MAPKK1b and ERK1c, respectively, by alternative splicing to generate a parallel signalling pathway. MAPK-ERK can phosphorylate MNK2, which is alternatively spliced and regulated by the oncogenic splicing factor SRSF1. SRSF1 upregulates a prooncogenic Mnk2b isoform and reduces Mnk2a isoform of this kinase. Blue: tumor suppressors, red: oncogenes, and green: parallel pathway.

Downstream to RTK activation, the small GTPase Ras is loaded with GTP and activated. Of the three genes encoding for Ras proteins (K-Ras, H-Ras, and N-Ras), K-Ras and H-Ras can include or exclude an exon termed IDX and generate p19- or p21-Ras, respectively [44]. p19-Ras cannot interact with A-Raf or Rin1, but binds RACK1 and may act in an antagonistic manner to p21-Ras [42]. Alternative splicing of the cytosolic kinase B-Raf, a downstream effector of the small GTPase Ras in the mitogen-activated protein (MAP) kinase pathway, gives rise to several isoforms devoid of the N-terminal autoinhibitory domain [20]. These constitutively active protein isoforms are able to activate the MAP kinase signalling pathway and to induce tumor formation in nude mice [20] (Figure 1, Table 1). Furthermore, alterations in the splicing profile of signalling components can also play a role in the acquisition of drug resistance. As an example, the mutant B-Raf(V600E) allele generates splicing isoforms that lack the Ras-binding domain and are able to dimerize in a Ras-independent manner [21]. Importantly, B-Raf(V600E) splice transcripts have been detected in several melanoma patients with acquired resistance to vemurafenib, an inhibitor of Raf activity used in clinical practice [21]. In addition, two truncated splicing isoforms of the cytosolic kinase A-Raf, another member of the Raf kinase family, negatively regulate A-Raf (Raf1) kinase activity by sequestering the upstream Ras GTPase activator [45, 46] (Figure 1). A-Raf_short_ which arises from retention of introns 2 and 4 is a 171-amino acid protein lacking two-thirds of the C-terminal region including the kinase domain [46]. In human head and neck carcinomas c-myc can induce high levels of the splicing regulator hnRNP H, which in turn increases expression of the full-length A-Raf protein, while decreasing the expression of A-Raf_short_ [46]. Another truncated isoform is DA-Raf1, which contains a premature termination codon, due to intron 6 retention. This isoform comprises of only the N-terminal Ras-binding domain of A-Raf [45]. Its overexpression impairs the tumorigenic potential of mutant K-Ras transformed mouse NIH3T3 fibroblasts in nude mice [45]. Although identified in murine cells, it can also affect the activity of human A-Raf. In a similar fashion, B-Raf truncated splicing variants devoid of kinase activity have been recently identified in colorectal cancer cells [40]. These truncated B-Raf splicing variants are generated through several alternative splicing events, such as skipping of exons 14-15 or inclusion of one of the additional exons 15b, 16b, and 16c, resulting in premature stop codons [40].

 The Ras/Raf/MAPK cascade can be activated by the epidermal growth factor receptor (EGFR/ErbB1), a member of the ErbB receptor tyrosine kinase family, which is frequently mutated and overexpressed in different human cancers, including glioma, non-small-cell lung carcinoma, ovarian carcinoma, and prostate carcinoma [47]. The most studied EGFR variant is the type III epidermal growth factor receptor mutant EGFRvIII (also referred to as ΔEGFR or de2-7 EGFR), containing an inframe deletion of exons 2–7 that can be generated either by gene rearrangement or altered pre-mRNA processing [48, 49]. EGFRvIII lacks a portion of the extracellular ligand-binding domain, is constitutively active in a ligand-independent manner, and confers growth advantage to cancer cells [49, 50] (Figure 1). The selective expression of EGFRvIII in several tumors, but not in normal tissues, makes it an extremely attractive target for anticancer therapy [50]. Another splicing isoform of EGFR, called de4 EGFR, is produced by skipping of exon 4. Similar to EGFRvIII, de4 EGFR undergoes ligand-independent activation and self-dimerization and displays transformation capabilities as well as metastasis-promoting potential [51]. Not only is de4 EGFR detectable in several human tumors, including glioma, prostate, and ovarian, but also its expression correlates with the malignant degree of glioblastomas [51]. 

 A fascinating example of the link between alternative splicing and signalling cascades has been recently provided by studying the metabolic effects, increased glucose uptake and lactate production, of EGFR activation in human cancer cells. The ability of EGFR activation to modulate the metabolism of cancer cells requires the expression of the PKM2 splicing isoform of pyruvate kinase M (PKM) [52], the enzyme that catalyzes the final step of glycolysis. There are two variants of PKM which are generated through alternative splicing of two mutually exclusive exons: exon 9 included in *PKM1* transcripts and exon 10 in *PKM2*. The choice between exon 9 and 10 is controlled by polypyrimidine tract binding protein (PTB, also known as PTBP1 or hnRNP I) and hnRNP A1/A2 that bind to sequences flanking exon 9, thus inhibiting the selection of exon 9 and promoting exon 10 inclusion [53, 54]. EGFR activation acts at different levels in the expression of PKM2 by increasing transcription of both* PTB* and *PKM* genes. Yang and collaborators have recently reported that EGFR upregulation of PKM2, but not PKM1, requires NF-*κ*B activation, which is mediated by PLC*γ*1 and PKC*ε* monoubiquitylation-dependent IKKbeta activation [52, 55]. Moreover, RNAi-mediated knock-down experiments indicate that PTB mediates the effect of EGFR on splicing of the *PKM* gene but not on transcription. Thus, a coordinated transcription-splicing program controlled by EGFR activation is responsible for the expression of the PKM2 isoform and for the distinctive metabolic features of cancer cells. 

 Another interesting example of regulation of the Ras-MAPK pathway by alternative splicing is the observation made by Cartegni's group that intronic polyadenylation, concomitantly (and in competition) with pre-mRNA splicing, can generate truncated soluble receptor tyrosine kinases (RTKs). These isoforms lack the anchoring transmembrane domain and the intracellular kinase domain and can act as dominant-negative regulators [28] (Figure 1). These secreted decoy receptors can shut down the relevant tumorigenic signalling pathways by titrating out the ligand or by trapping the wild-type receptors in nonfunctional heterodimers [28]. In particular, for the vascular endothelial growth factor receptor 2 (VEGFR2/KDR), the pivotal molecule in controlling VEGF-dependent functions, the expression of the dominant-negative sKDR strongly inhibited the angiogenesis process in both primary HUVEC endothelial cells and in the same cells exposed to conditioned media and simultaneously treated with VEGF-A. Thus, artificially increasing production of these truncated soluble receptors could be a valid approach to interfere with angiogenic paracrine and autocrine loops. 

 Soluble isoforms produced by alternative splicing and containing only the extracellular domain of the protein have also been detected for the EGFR/ErbB1 tyrosine kinase receptor [47, 56]. These truncated soluble EGFR variants have been detected in several cancer types, and their levels, circulating as well as in tumor tissues, have been used as prognostic and predictive markers for ovarian, cervical, lung, and breast cancers [57–59]. Alternative splicing of other tyrosine kinase receptors such as the insulin, IGF-1, FGF, ERBB2, and ERBB4 receptors can also modulate the activation of their downstream signalling pathways [2, 19, 29, 36, 60–65]. An additional example of alternative splicing in the Ras-MAPK pathway is the splicing of MEK1b and ERK1c [66, 67]. MEK1 (MAPKK1) phosphorylates and activates ERK1/ERK2 [66, 67]. MEK1b is a unique splicing isoform of MEK1 which specifically phosphorylates ERK1c, an isoform of ERK1 [66, 67]. Thus, by alternative splicing a parallel pathway is generated, and this confers higher substrate specificity to this branch of the pathway [66, 67] (Figure 1). Another component in the MAPK pathway which is regulated by alternative splicing is the kinase *MKNK2*, which is regulated by the oncogenic splicing factor SRSF1. Upregulation of SRSF1 downregulates the expression of the Mnk2a isoform and induces the expression of the Mnk2b isoform [14] (Figure 1). Mnk2b is a prooncogenic isoform that activates eIF4E independently of MAPK activation [14]. Recently, this splicing switch was shown to regulate the resistance of prostate cancer cells to chemotherapy [68]. Altogether, these examples of components of the Ras-MAPK pathway which are regulated by alternative splicing open up the exciting possibility of exploiting dominant-negative splicing isoforms for therapeutic purposes, as antioncogenic agents.

## 3. Regulation of the PI3K-mTOR Pathway by Alternative Splicing

**Figure 2:**
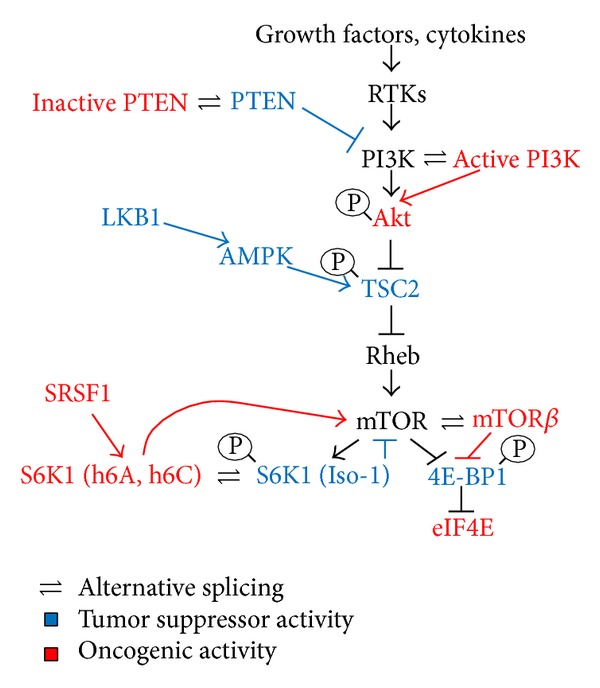
Alternative splicing regulation of the PI3K-mTOR pathway. Growth factors and cytokines activate receptor tyrosine kinases (RTKs) which in turn lead to activation of PI3K. PI3K phosphorylates phospholipids inducing the recruitment of Akt to the plasma membrane and its activation by PDK1. A splicing variant of the catalytic subunit of PI3K (p37 delta) is an active form that enhances PI3K activity. Akt phosphorylates and inactivates the tumor suppressor TSC2, which inhibits the small GTPase Rheb. GTP-bound Rheb can activate mTOR. mTOR*β* is an active splicing isoform of mTOR. mTOR phosphorylates S6K1 and 4E-PB1. 4E-BP1 phosphorylation induces its release from eIF4E, enhancing cap-dependent translation and malignant transformation. Oncogenic splicing factor SRSF1 can affect the alternative splicing of S6K1 inducing oncogenic short isoforms of this kinase (h6A, h6C) which bind mTOR and enhance 4E-BP1 phosphorylation and cap-dependent translation. Blue: tumor suppressors, red: oncogenes.

Upon RTK activation, both the Ras-MAPK and the PI3K-mTOR pathways are activated [16, 17] (Figure 2). Kinase PI3K, which phosphorylates 3,5, phosphoinositides, consists of a regulatory subunit, p85, and a catalytic subunit, p110. Both p85 and p110 undergo alternative splicing which can modulate the activity of PI3K [69, 70] (Figure 2). The tumor suppressor phosphatase PTEN, which counteracts PI3K activity, is also regulated by alternative splicing, and splicing isoforms of PTEN that are inactive and act in a dominant-negative fashion have been detected in Cowden syndrome and in breast cancer [71]. Upon production of phosphoinositide by PI3K, the kinase Akt is recruited to the plasma membrane and is activated by PDK1 [17]. Active Akt phosphorylates and inactivates the tumor suppressor complex of TSC1/TSC2. Both TSC1 and TSC2 tumor suppressors are alternatively spliced although inactivation by alternative splicing has not been demonstrated [72–74]. TSC1/2 inactivates the GTPase Rheb, a small GTPase from the Ras protein family which binds and activates mTOR [18]. Alternative splicing of Rheb is not known. mTOR can undergo alternative splicing to generate an activated form called mTORbeta which is oncogenic, although its regulation and role in human cancer have yet to be demonstrated [75]. Recently it was shown that the splicing factor Sam68 controls alternative splicing of mTOR, reducing the retention of intron 5. Cells from Sam68 knockout mice display reduced levels of mTOR mRNA due to nonsense-mediated decay degradation of the intron-retained transcript and reduced activity of the mTOR pathway [76]. mTOR phosphorylates and activates several substrates. Among the best-characterized mTOR substrates are S6K1, which phosphorylates the ribosomal protein S6 and regulates the translation process [18], and eIF4E-BP1, which is involved in the formation of an active translation initiation complex [14, 18, 77]. Alternative splicing of *S6K1* transcripts is controlled by the oncogenic splicing factor SRSF1 [14]. Specifically, SRSF1 promotes the production of short S6K1 isoforms, frequently upregulated in breast cancer cell lines and tumors [14, 78]. These variants display oncogenic properties as they are able to enhance cell transformation, motility, and anchorage-independent growth of breast epithelial cells [78]. The short splicing variants of S6K1 are not substrates of the signalling cascade (Akt/mTOR) but generate a signal loop to activate the mTOR pathway in the absence of external stimuli. Indeed, S6K1 short isoforms are able to bind and increase mTORC1 activity, leading to 4E-BP1 inactivation and enhancing translation of several oncogenes and antiapoptotic genes [78] (Figure 2).

## 4. Extracellular Stimuli Targeting Components of the Splicing Machinery

Although for a long time transcription factors have been considered the ultimate effectors of the signalling cascade pathways, in the last few years splicing machinery components and alternative splicing regulatory proteins have also been recognized as important targets [79]. The activity of splicing factors is normally regulated through posttranscriptional modifications, mainly phosphorylation [80], and therefore it is not surprising that signalling cascades are involved in misregulation of splicing factors. 

 The PI3K/Akt/mTOR signalling cascade, frequently altered in malignancies [81], has been found to affect the alternative splicing profile of cancer-relevant genes via Akt-dependent phosphorylation of several SR proteins, such as SRSF1 and SRSF5 [82–84]. Alternative splicing factor 45 (SPF45) has also been identified as a target of multiple MAP kinases, such as ERKs (extracellular signal-regulated kinases), JNKs (Jun N-terminal kinases), and p38 MAPK [85]. Interestingly, phosphorylation of SPF45 impacts cell proliferation and cell adhesion programs, through downregulation of the human epidermal growth factor receptor (ErbB2) and regulation of the alternative splicing of the *fibronectin 1* gene (*FN1*), respectively [85].

Signals from the EGF receptor, which activate the PI3K-Akt pathway, induce phosphorylation of the SR protein kinase SRPK1 which in turn phosphorylates SR proteins, such as SRSF1. Phosphorylation of SRSF1 leads to its activation and modulation of the cell's alternative splicing program [86]. The complete landscape of alternative splicing changes by such signals has yet to be elucidated.

Stress signals emanating from osmotic shock and other modes of stress which activate the MEK4-/MEK7-p38-MAPK pathway control cell localization of the splicing factor hnRNP A1 [87–89]. Activation of the p38-MAPK pathway induces hnRNP A1 phosphorylation and export from the nucleus into the cytoplasm [87, 89]. Reducing nuclear hnRNP A1 levels in response to stress is expected to affect many alternative splicing events which have yet to be determined.

**Figure 3:**
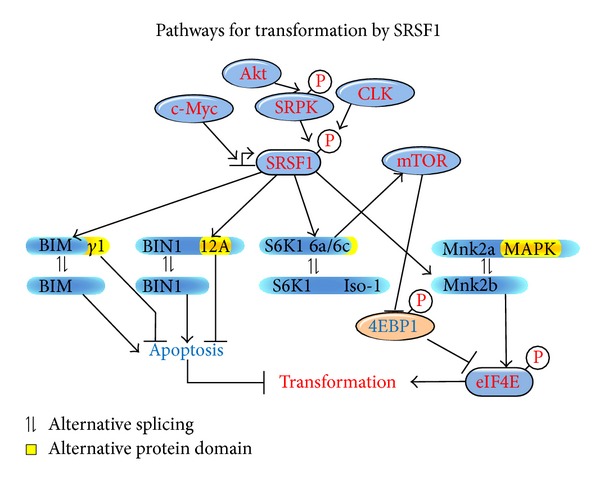
Pathways for transformation by SRSF1. SRSF1 is a transcriptional target of the c-myc protooncogene and can be phosphorylated by SRPK or CLK downstream to Akt. SRSF1 alters the splicing of BIM, BIN1, S6K1, and Mnk2 regulating the mTOR and MAPK pathways, increasing translation, and inhibiting apoptosis.

 A very complex and intriguing story has emerged from the study of the multiple functions of SRSF1. SRSF1 is a splicing factor with an oncogenic activity that derives from its involvement in the Akt-mTOR pathway [14, 90] (Figure 3). The Akt-dependent phosphorylation of SRSF1 affects its function in splicing regulation and translation. Thus, for instance, upon phosphorylation by Akt, SRSF1 enhances the production of EDA-FN, a splicing isoform of *fibronectin 1 *(*FN1*) expressed in various malignancies but undetectable in normal tissues [82, 91]. SRSF1 phosphorylation by Clk (CLK1-4) or SRPK kinases (SRPK1-2) has the opposite effect on *EDA-FN* splicing, pointing to SRSF1 as a sort of integrator component that can switch between splicing decisions in response to various external cues [91]. This view is further supported by the observation that the effect of SRSF1 on *EDA-FN* splicing is abrogated by rapamycin, an inhibitor of mTORC1 (mTOR complex-1) [82]. SRSF1 is not only a target but also an effector of signalling cascades. Indeed, it is able to activate mTORC1 leading to downstream phosphorylation of two of its substrates; S6K1 kinase and 4E-BP1 [77, 78, 90]. The mechanism by which SRSF1 activates mTORC1 still needs to be clarified. However, mTORC1 activation by SRSF1 bypasses upstream PI3K/Akt signalling and is essential for SRSF1-mediated transformation in nude mice, suggesting that mTOR inhibitors may be useful for treatment of tumors with SRSF1 upregulation [78, 90]. 

## 5. Conclusions

Our current knowledge is only the tip of the iceberg in terms of the many mechanisms by which alternative splicing regulates the Ras-MAPK and the PI3K-mTOR signalling pathways. This is also true for the mechanisms by which these signalling cascades regulate the activity of splicing regulators such as SR and hnRNP proteins leading to modulation of the cell's splicing landscape. The diagnostic and therapeutic potential of manipulating the Ras-MAPK and the PI3K-mTOR pathways, by modulating alternative splicing of components of these pathways, is immense. Detection of alternatively spliced isoforms of key substrates, such as S6K1, might be used to predict the sensitivity of tumors to mTOR inhibitors, enabling better choices of drug treatment in the clinic [78]. Another clinical application concerns resistance of tumors to drugs which, as in the case of B-Raf splicing, can arise by alterations in alternative splicing. Elimination of a drug-binding domain, for example, does not require a genomic change but rather can occur by skipping of the relevant exon, resulting in a new splicing isoform which is drug resistant. Even with all these new findings there still remains a lot to be learned about how alternative splicing regulates signalling and how signalling regulates splicing. This field holds great promise for advancing our understanding of the cancer process and for use in the clinic.

## Figures and Tables

**Table 1:** Alternative splicing of Ras-MAPK and PI3K-mTOR signaling components.

Signalling component	Gene name	Splicing isoform activity	Type of cancer	Reference number
RTK	EGFR	Constitutively active receptor/soluble decoy isoform, enhanced signalling, survival, and tumorigenicity.	Glioblastoma, lung	[28, 47–49, 51, 56–58]
RTK	RON	Constitutively active receptor, enhanced signalling, invasion, and motility.	Glioblastoma, colon, breast, and gastric	[34, 35]
RTK	MET	Constitutively active receptor/soluble decoy isoform, enhanced/reduced signalling, invasion, and motility.	Ovarian, lung, and HCC	[38, 39]
RTK	FGFR	Induction of EMT, invasion, and motility.	Prostate, pancreatic, and breast	[28, 29, 36, 61]
RTK	INSR	Differential ligand binding (IGF-II) and oncogenic activity.	HCC, thyroid, and ovarian	[31, 37]
RTK	VEGFR	Soluble decoy isoform, enhanced/reduced angiogenesis, and survival.	Lung, breast	[28, 32]

Cytosolic kinase	Fyn	Enhanced/reduced kinase activity, survival of epithelial cells.	Unknown	[43]
Cytosolic kinase	mTOR	Constitutively active kinase, oncogenic activity.	HCC	[75]
Cytosolic kinase	S6K1	Tumor suppressor/oncogenic isoforms, activates/inhibits mTORC1.	Breast, lung	[14, 78]
Cytosolic kinase	A-Raf	Enhanced/reduced binding to Ras and activation of the MAPK pathway.	HCC, head, and neck	[45, 46]
Cytosolic kinase	B-Raf	Enhanced/reduced kinase activity, activation of the MAPK pathway, and resistance to BRAF kinase inhibitors.	Colon, melanoma	[20, 21, 40]
Cytosolic kinase	MEK1	Alternative pathway with a different substrate.	Unknown	[66, 67]
Cytosolic kinase	ERK1	Alternative pathway with different substrates.	Unknown	[66, 67]
Cytosolic kinase	MKNK2	Oncogenic isoform that enhances eIF4E phosphorylation and a tumor-suppressive isoform.	Lung, breast, colon, and pancreas	[14, 68]

Phospholipid phosphatase	PTEN	Active/inactive tumor suppressor.	Unknown	[71]
Phospholipid kinase	PI3K	Constitutively active kinase, enhanced downstream signalling.	Unknown	[69, 70]

Small GTPase	Ras	Enhanced/reduced binding to Raf and Rin and activation of the MAPK pathway.	Unknown	[32, 44]
GTPase activator (GAP)	TSC2	Inactivation of a tumor suppressor.	Tuberous sclerosis	[72–74]
